# Etiologies of olfactory dysfunction in a pediatric population: based on a retrospective analysis of data from an outpatient clinic

**DOI:** 10.1007/s00405-020-06087-4

**Published:** 2020-06-01

**Authors:** Valentin Alexander Schriever, Thomas Hummel

**Affiliations:** 1 Abteilung Neuropädiatrie an der Klinik und Poliklinik für Kinder- und Jugendmedizin, Medizinische Fakultät Carl Gustav Carus, Technische Universität Dresden, Fetscherstrasse 74, 01307 Dresden, Germany; 2 Smell and Taste Clinic, Department of Otorhinolaryngology, Medizinische Fakultät Carl Gustav Carus, Technische Universität Dresden, Dresden, Germany

**Keywords:** Olfactory dysfunction, Children, Epidemiology of olfactory dysfunction, Smell loss

## Abstract

**Purpose:**

Although the prevalence of olfactory dysfunction in children is thought to be lower compared to adults, little is known about the actual frequency of etiologies of smell dysfunction in children. Aim of the study was (i) to describe the epidemiology of olfactory dysfunction in a pediatric population and (ii) to compare the distribution of etiologies to adults.

**Material and methods:**

Data of patients consulting a smell and taste clinic between 2000 and 2017 were retrospectively analyzed. Frequency of major causes of olfactory dysfunction was examined with a focus on the pediatric population.

**Results:**

A total of 7153 patients (164 children) were included in the analysis. Most children presented with congenital olfactory dysfunction (67%), or head-trauma (12%). In contrast, the cumulative frequency of olfactory loss associated with sinonasal disorders or acute infections of the upper airways was 6%. The frequency of etiologies of olfactory dysfunction changed with age: While the frequency of patients with congenital anosmia decreased, the frequency of causes related to infections of the upper respiratory tract and idiopathic causes increased.

**Conclusion:**

About 2/3 of olfactory dysfunction in children are congenital while 1/3 is acquired. The frequency of etiologies causing olfactory dysfunction change significantly from children to an adult population.

## Introduction

Although olfactory dysfunction, its etiologies and possible implications on a pediatric population have gained more interest in recent years, little is known about these topics. The prevalence of olfactory dysfunction in children is thought to be lower compared to adults, and especially older adults [1]. No study has been conducted so far to systematically evaluate the prevalence of olfactory dysfunction in a pediatric population. Etiologies of olfactory dysfunction can be divided into congenital [2] and acquired [3] etiologies of olfactory dysfunction. The frequency of the different etiologies in a pediatric population is unknown.

Aim of this short communication is twofold: (i) describe the epidemiology of olfactory dysfunction in a pediatric population seeking medical consultation, (ii) compare the distribution of etiologies between children and adults.

## Material and methods

Retrospective analysis of consecutive patients visiting a specialized smell and taste clinic between 2000 and 2017. Olfactory testing was assessed using the “Sniffin’ Sticks” battery including evaluation of olfactory threshold, odor discrimination and odor identification (TDI). Diagnosis of olfactory dysfunction was based on published reference values [4]. To answer our questions, the study population was separated into five age groups (i) < 18 years, (ii)18–39 years, (iii) 40–59 years, (iv) 60–79 years and (v) > 80 years. The pediatric population was additionally split into two age groups (P1) 5–11 years (*n* = 49) and (P2) 12–17 years (*n* = 115).

Etiologies responsible for at least 5% of olfactory dysfunction in any age group were listed as main causes for olfactory dysfunction [congenital, idiopathic, neurodegenerative, head trauma, sinonasal and upper respiratory tract infection (URTI)]. All other etiologies (e.g. chemotherapy, radiation, iatrogenic) were grouped in the category “others”.

## Results

**Fig. 1:**
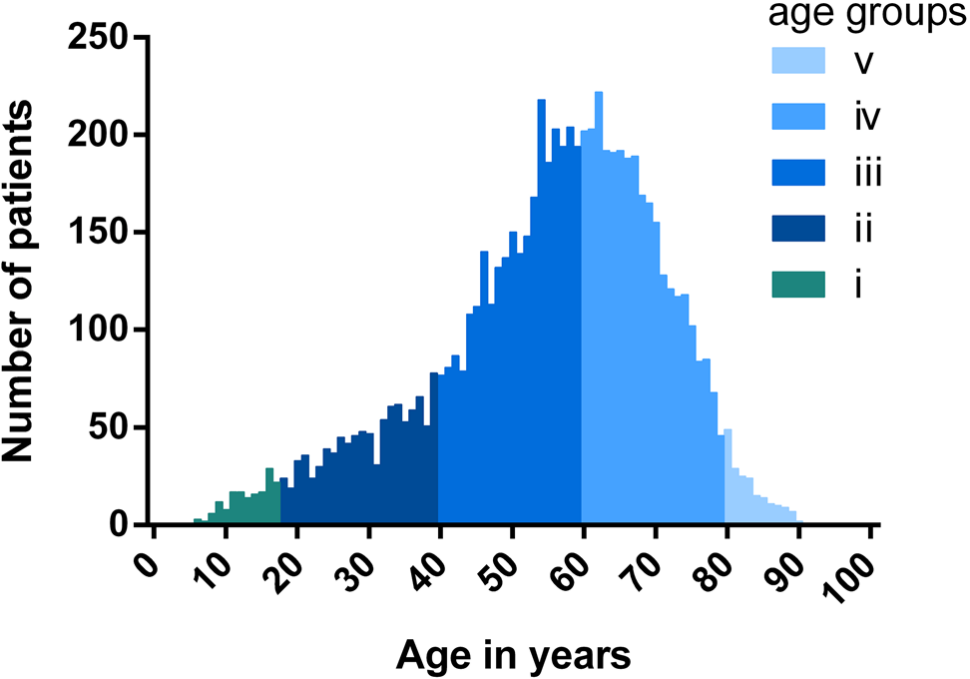
Age distribution of study population. The five age groups are shown in different colors (i: ≤ 17 years, ii: 18–39 years, iii: 40–59 years, iv: 60–79 years, ≥ 80 years)

**Fig. 2:**
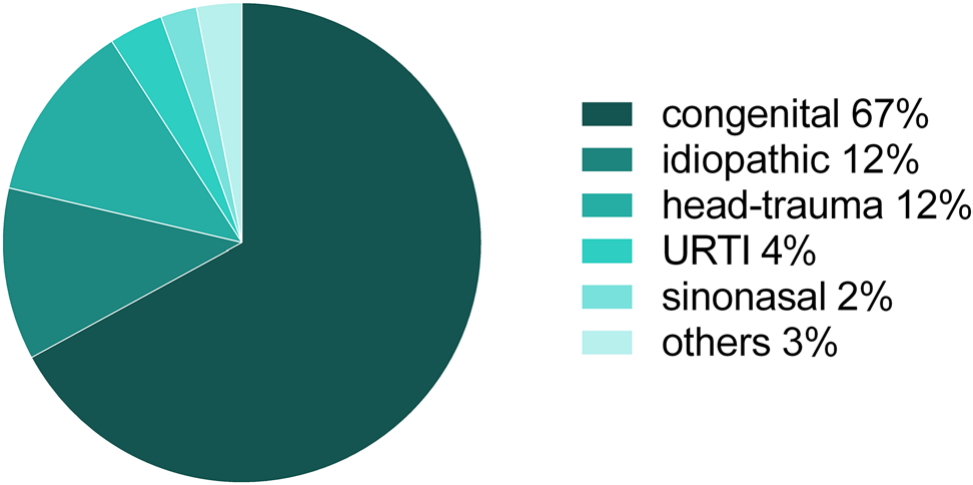
Etiologies of olfactory dysfunction in children. Frequency of etiologies of olfactory dysfunction in a pediatric population. Congenital olfactory dysfunction is responsible for the majority of cases. *URTI* upper respiratory infection

A total of 7153 patients were included in the analysis. The age distribution of the study population is shown in Fig. 1, visualizing that the majority of patients seeking medical consultation were between 40–80 years old. The pediatric population consisted of 164 children and adolescents (58% girls, 42% boys), mean age 13.2 ± 3.0 years (range 5–17 years), accounting for 2.3% of the whole study population. Full “Sniffin’ Sticks” TDI scores including olfactory threshold, odor discrimination and odor identification testing was conducted in 144 children resulting in a mean TDI score of 13.77 ± 6.24 points (range 3.0–30.5 points). Slightly higher scores were achieved in the adolescents (P2) (TDI: 14.02 ± 6.52 points) compared to younger children (P1) (TDI: 12.98 ± 5.31 points). Most children presented with congenital olfactory dysfunction (67%), followed by head-trauma (12%) and idiopathic (12%) olfactory dysfunction. Only 4% and 2% of olfactory dysfunction were due to URTI and sinonasal causes in this age group. Causes of olfactory dysfunction in the pediatric population are shown in Fig. 2. In addition, TDI scores (mean ± SD) are displayed for the different etiologies of olfactory dysfunction for the pediatric study population in Table 1. In our study population, 21% of patients had an acquired olfactory dysfunction. An idiopathic cause was diagnosed in 12% of the cases. Because idiopathic are most likely to relate to acquired olfactory dysfunction, this increases this category to 33% of our patients. Further analysis showed, that the frequencies of the three major causes of olfactory dysfunction in the pediatric population (congenital olfactory dysfunction, head-trauma and idiopathic causes) did not differ between younger (P1) and older (P2) children (*Χ*^2^ = 0.69, *p* = 0.71).

**Table 1:** Olfactory test results for the pediatric population

Etiology of olfactory dysfunction	Frequency of etiology (in %)	TDI (mean ± SD)
Congenital	67	11.53 ± 4.59
Idiopathic	12	16.41 ± 5.91
Head-trauma	12	19.84 ± 7.35
URTI	4	18.20 ± 8.25
Sinonasal	2	16.83 ± 5.82
Other	3	19.06 ± 7.05

**Fig. 3:**
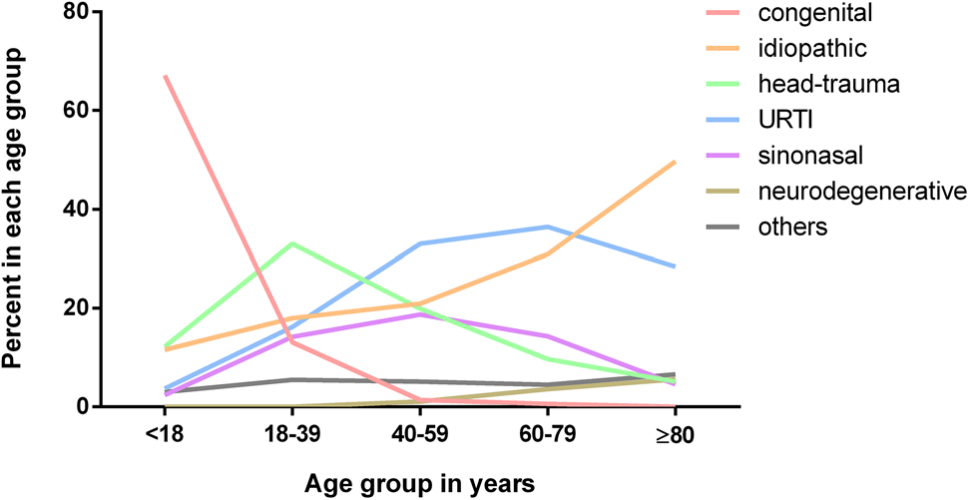
Etiologies of olfactory dysfunction change with age. Age-related distribution of etiologies of olfactory dysfunction. While the percentage of patients with congenital olfactory dysfunction seeking medical consultation decreases, other etiologies such as URTI increases with age (i: ≤ 17 years, ii: 18–39 years, iii: 40–59 years, iv: 60–79 years, ≥ 80 years)

The frequencies of etiologies of olfactory dysfunction differed significantly between the five age groups (*Χ*^2^ = 2605, *p* < 0.001). Figure 3 displays the percentage of causes of olfactory dysfunction for each age group. The frequency of patients seeking medical consultation due to congenital olfactory dysfunction dramatically decreases with age while other causes (i.e., URTI-related olfactory loss and idiopathic causes) increase with increasing age. The frequency of etiologies of olfactory dysfunction such as sinonasal and other causes remains stable in adult patients over a wide age range.

## Discussion

Pediatric patients accounted for 2.3% of our study population. This is in agreement with previously reported 2% of pediatric patients in a smell and taste clinic [5]. To our knowledge only one publication, a case series of 37 children, described the frequencies of etiologies in pediatric olfactory dysfunction [6]. The diagnosis of olfactory dysfunction was based on self-reports. Unfortunately, standardized olfactory testing was only performed in a minority of these patients [6], although self-reports on olfactory function appear to be notoriously difficult to interpret (e.g. [7]). Therefore, this is the first study to report etiologies of olfactory dysfunction and their frequency in a larger pediatric population.

Congenital olfactory dysfunction was diagnosed in two-thirds of our pediatric population. Congenital olfactory dysfunction or congenital anosmia includes a wide range of etiologies with two main groups—isolated and syndromic forms of olfactory dysfunction [2]. The term does not define the actual cause of olfactory dysfunction but rather its onset. About one-third of pediatric patients were diagnosed with an acquired olfactory dysfunction with head-trauma as one of the main etiologies. Several studies have previously shown the association between traumatic brain injury and olfactory dysfunction in children [8].

Although previous studies describing the causes of olfactory dysfunction in adults differ in the reported prevalence of the main etiologies [9], several studies reported an age dependency. The prevalence of olfactory dysfunction due to head-trauma has been reported to decrease with age, while olfactory dysfunction due to URTI increases [10, 11]. Our study confirms these findings in a very large population of more than 7000 patients.

Olfactory dysfunction caused by sinonasal etiologies and URTI occur only rarely in a pediatric population. At least for URTI this might be due to a higher regeneration capacity of olfactory receptor neurons at a younger age [12] and the cumulative damaging effect of repeated infections in older age. This is in contrast to Hauser et al. who reported rhinologic diseases as the most common etiology for olfactory dysfunction in their pediatric population [6]. Several reasons might account for this difference. (1) Olfactory testing was only conducted in the minority of cases reported by Hauser et al., (2) the sample size of the current study is larger compared to the sample size presented by Hauser et al., and (3), most importantly, the frequencies of etiologies of olfactory dysfunction is influenced by the investigated patient population. Patients included in our study actively sought medical consultation due to subjective olfactory loss.

The present results do not allow a statement about the overall prevalence of olfactory dysfunction in children and adolescence. However, for the first time this short communication describes the frequencies of etiologies of olfactory dysfunction in a larger pediatric population of a specialized smell clinic. The possibility of acquired olfactory dysfunction should be considered in the diagnostic work-up. In addition, further studies are needed to better understand the underlying mechanisms of congenital olfactory dysfunction.
