# Correction to: Etiologies of olfactory dysfunction in a pediatric population: based on a retrospective analysis of data from an outpatient clinic

**DOI:** 10.1007/s00405-021-06961-9

**Published:** 2021-07-05

**Authors:** Valentin Alexander Schriever, Thomas Hummel

**Affiliations:** 1 Abteilung Neuropädiatrie an der Klinik und Poliklinik für Kinder- und JugendmedizinMedizinische Fakultät Carl Gustav Carus, Technische Universität Dresden, Fetscherstrasse 74, 01307 Dresden, Germany; 2 Smell and Taste Clinic, Department of Otorhinolaryngology, Medizinische Fakultät Carl Gustav Carus, Technische Universität Dresden, Dresden, Germany

## Correction to: European Archives of Oto-Rhino-Laryngology (2020) 277:3213–3216 https://doi.org/10.1007/s00405-020-06087-4

The article “Etiologies of olfactory dysfunction in a pediatric population: based on a retrospective analysis of data from an outpatient clinic”, written by Valentin Alexander Schriever and Thomas Hummel, was originally published Online First without Open Access. After publication in volume 277, issue 11, pages 3213–3216 the author decided to opt for Open Choice and to make the article an Open Access publication. Therefore, the copyright of the article has been changed to © The Author(s) 2021 and the article is forthwith distributed under the terms of the Creative Commons Attribution 4.0 International License, which permits use, sharing, adaptation, distribution and reproduction in any medium or format, as long as you give appropriate credit to the original author(s) and the source, provide a link to the Creative Commons licence, and indicate if changes were made. The images or other third party material in this article are included in the article’s Creative Commons licence, unless indicated otherwise in a credit line to the material. If material is not included in the article’s Creative Commons licence and your intended use is not permitted by statutory regulation or exceeds the permitted use, you will need to obtain permission directly from the copyright holder. To view a copy of this licence, visit http://creativecommons.org/licenses/by/4.0. Open access funding enabled and organized by Projekt DEAL.

The original article has been corrected.

